# *HNF1α*-Q125ter-mediated mitochondrial dysfunction and impaired mitophagy in β-cells

**DOI:** 10.1530/JME-25-0033

**Published:** 2025-11-10

**Authors:** Fei Jiang, Jie Huang, Xinyan Chen, Xiao-Xi Zhang, Yinling Chen

**Affiliations:** ^1^ School of Medicine, Hangzhou City University, Hangzhou, China; ^2^ Anji People’s Hospital, Affiliated Anji Hospital, School of Medicine, Hangzhou City University, Huzhou, China; ^3^ Center of Diabetic Systems Medicine, Guangxi Key Laboratory of Excellence, Guilin Medical University, Guilin, China; ^4^ Fujian Provincial Key Laboratory of Innovative Drug Target Research, School of Pharmaceutical Sciences and School of Life Sciences, Xiamen University, Xiamen, China

**Keywords:** *HNF1α*-Q125ter, mitochondrial function, mitophagy, mTOR/p70S6K pathway

## Abstract

Maturity-onset diabetes of the young (MODY) is a form of monogenic diabetes caused by single-gene mutations. MODY3, the most common subtype, results from mutations in the hepatocyte nuclear factor 1-alpha (*HNF1α*) gene. *HNF1α* is a transcription factor essential for pancreatic β-cell function and insulin production. Clinically, β-cells in MODY3 patients generally retain intact sulfonylurea receptor function, making sulfonylureas the preferred treatment. However, a novel loss-of-function variant, *HNF1α*-Q125ter, has been shown to induce sulfonylurea insensitivity in MODY3 patients. This study aimed to investigate the role and mechanism of *HNF1α*-Q125ter-mediated mitochondrial dysfunction and impaired mitophagy in new variant-induced β-cell dysfunction. Mitophagy-related protein and transcription levels were analysed by Western blotting and reverse transcription-quantitative PCR (RT-qPCR). Mitochondrial morphology was examined by transmission electron microscopy. Ins-1 cells were transfected with overexpression constructs for *HNF1α*-Q125ter or short hairpin RNA targeting *HNF1a* (*shHNF1α*) to assess its effects on mitochondrial function and mitophagy. Ins-1 cells expressing *HNF1α*-Q125ter showed decreased mitochondrial number, oxygen consumption, and energy metabolism. Correspondingly, mitochondrial morphology was damaged in an *hnf1a^+/−^* zebrafish model. *HNF1α*-Q125ter also inhibited mitophagy by suppressing the mRNA expression of PTEN-induced kinase 1 (PINK1), pyruvate dehydrogenase E1 subunit α1 (PDHA1), and Parkin RBR E3 ubiquitin-protein ligase (Parkin). Mechanistically, *HNF1α*-Q125ter impaired autophagy by downregulating phosphorylated mammalian target of rapamycin (p-mTOR) (Ser2448) and phosphorylated-70 kDa ribosomal protein S6 kinase (p-p70S6K) (Thr389). In conclusion, our findings suggest that *HNF1α*-Q125ter induces mitophagy dysfunction by suppressing the p-mTOR(ser2448)/p-p70S6K(Thr389) signalling pathway, providing novel insights into the mechanisms underlying sulfonylurea insensitivity in patients with this variant.

## Introduction

Diabetes mellitus is a global pandemic that places a significant burden on healthcare systems worldwide and results from both acquired and genetic factors (Qian *et al.* 2023). Research on monogenic diabetes, such as maturity-onset diabetes of the young (MODY), has advanced our understanding of the genetic basis of diabetes (Zhang *et al.* 2021). Among these forms, hepatocyte nuclear factor 1-alpha (*HNF1A*)-MODY, the most prevalent form, arises from mutations in the HNF1A gene, which encodes the transcription factor *HNF1α* (Riddle *et al.* 2020). Although the association between *HNF1A* deficiency and diabetes is well established, the precise mechanisms by which *HNF1α* regulates mature human islet cell function remain unclear.

Important findings have come from research on patients with HNF1A-MODY, who show a distinctive insulin secretion defect that usually responds well to sulfonylurea treatment (Bacon *et al.* 2016). However, our earlier study revealed that the *HNF1α*-Q125ter mutation confers sulfonylurea insensitivity in MODY3 patients and leads to β-cell dysfunction by triggering endoplasmic reticulum (ER) stress (Chen *et al.* 2022).

Mitochondria are essential for the release of insulin. Insulin is released when the ATP/ADP ratio rises due to glucose-derived mitochondrial metabolism (Colclough *et al.* 2013). Glucotoxicity-induced β-cell failure is caused by a variety of factors, including alterations in mitochondrial dynamics (Yoon *et al.* 2011), decreased mitochondrial respiration (Barlow & Affourtit 2013), altered mitochondrial structure (Anello *et al.* 2005), and decreased ATP generation (Masini *et al.* 2014). Furthermore, peptides expressed by the mitochondria, including Humanin, which is a sign of oxidative stress, improve insulin sensitivity, encourage the survival of β-cells, and postpone the onset of diabetes (Ikonen *et al.* 2003, Lee *et al.* 2013, Miller *et al.* 2022).

Another mitochondrial peptide, mitochondrial open reading frame of the twelve S rRNA-c (MOTS-c), has been shown to delay insulin resistance and reduce obesity in mice (Lee *et al.* 2015). Kong *et al.* further demonstrated that MOTS-c prevents islet cell senescence and delays diabetes progression through an mTOCR1-dependent signalling pathway (Kong *et al.* 2025). Conversely, mitochondrial damage can lead to excessive production of mitochondrial reactive oxygen species (mtROS) (Deng *et al.* 2021.), depletion of mitochondrial DNA (mtDNA) (Lampert *et al.* 2019), and impaired intracellular calcium storage (Lombardi *et al.* 2017). These metabolic disturbances may alter the expression of downstream targets of *HNF1a*, impair insulin secretion, and accelerate disease progression in HNF1A-MODY patients. Recent studies have shown that dominant-negative mutations in HNF1α impair mitochondrial structure, glucose oxidation, ATP synthesis, and hyperpolarisation (Kirkpatrick *et al.* 2011, Qian *et al.* 2023), suggesting that mitochondrial dysfunction may play a significant role in *HNF1α*-Q125ter-induced β-cell dysfunction. Nevertheless, the effects of *HNF1α*-Q125ter on β-cell mitochondria remains poorly characterised. Therefore, the roles of *HNF1α*-Q125ter in maintaining functions of mitochondria in β-cells have not been firmly established.

The challenges of examining the impact of novel *HNF1α* mutations on β cell mitochondria are emphasised by this ignorance. To address this challenge, we generated a heterozygous *HNF1α*-Q125ter variant in zebrafish (*hnf1a^+/−^*) using CRISPR/Cas9. In addition, we conducted *HNF1a* knockdown and *HNF1α*-Q125ter overexpression in Ins-1 cell lines. Using both *in vivo* and *in vitro* models, we investigated the mechanisms underlying mitochondrial dysfunction induced by *HNF1α*-Q125ter. Our findings revealed that *HNF1α*-Q125ter impairs mitochondrial function and disrupts mitophagy by inhibiting the mTOR/p70S6K signalling pathway.

## Materials and methods

### Zebrafish maintenance

Zebrafish (Danio rerio) were maintained in a recirculating aquaculture system (Haisheng, China). Embryos were collected and cultivated following established protocols (Kimmel *et al.* 1995). Transgenic lines employed in this study included Tg(-1.2ins:H2BmCherry) and Tg(gcga:GFP) (Zecchin *et al.* 2007, Maddison & Chen 2012). All animal experiments were performed in strict compliance with the guidelines and regulations established by the Xiamen University Institutional Animal Care and Use Committee (approved protocol XMULAC20160089, approved 10 March 2016).

### CRISPR/Cas9-mediated generation of zebrafish with *hnf1a* mutations

Guide RNA (gRNA) targeting HNF1α was designed using established methodologies (Xu *et al.* 2022). A 19-nucleotide target sequence (ACAACCTTCCCCAGAGAG) was selected through the CRISPR Scan web-based design platform. *In vitro* transcription of single gRNA (sgRNA) was executed with the MAXIscript T7 Transcription Kit (Invitrogen, USA). Embryos at the zygotic stage received simultaneous microinjection of synthesised sgRNA and recombinant Cas9 protein (New England Biolabs, China).

Founder (F0) mutants were matured and outcrossed with wild-type AB zebrafish to generate the F1 progeny. Genomic DNA obtained from caudal fin biopsies was amplified with primers (F: ATG​CTT​CAC​AAG​TAC​ATA​ATA​CA; R: TTG​AGG​TGC​TGC​GAC​AGA​T) and analysed by dual-matrix electrophoresis (1% agarose and polyacrylamide gels). Sanger sequencing of PCR products identified an individual carrying a biallelic two-base pair deletion in the targeted genomic region.

### Cell culture and transfection

Cellular experiments were adapted from established protocols (Chen *et al.* 2022). Ins-1 832/13 cells were cultured in RPMI-1640 medium (Invitrogen, USA) supplemented with 10% heat-inactivated foetal bovine serum (FBS) (Gibco, USA), 1 mM sodium pyruvate (Hyclone Laboratories, USA), 10 mM HEPES (Hyclone Laboratories, USA), 50 μM β-mercaptoethanol (Sigma, USA), and antibiotics (100 U/mL penicillin and 100 μg/mL streptomycin) (Sigma, USA). Cells were maintained at 37°C under a 5% CO_2_ atmosphere and passaged with 0.25% trypsin–EDTA at 70–80% confluence.

Plasmids used for transfection included pIRES2-eGFP (control vector), pIRES2-HNF1a-Q125ter-eGFP (truncated variant), pIRES2-HNF1a-eGFP (wild-type), shScramble (pPLK/GFP + Puro), and shHNF1a (pPLK/GFP + Puro mHNF1a shRNA). All plasmids were purchased from the Public Protein/Plasmid Library. Transfections were executed with Lipofectamine 3000 reagent (Invitrogen, Cat# L3000015), and cells were harvested 24 h post-transfection for downstream analyses.

### Islet isolation protocol

Pancreatic islets were extracted from larval zebrafish through enzymatic dissociation based on published methods (Chen *et al.* 2022). Larvae (wild-type or *hnf1a* mutants) were anaesthetised with tricaine, then incubated with 250 μL collagenase P (0.6 mg/mL in Hank’s balanced salt solution (HBSS), Roche, Switzerland) for 5 min at 37°C. The enzymatic reaction was quenched with 1 mL HBSS (Gibco, USA) containing 10% FBS (Gibco, USA). After centrifugation (300 *g*, 4°C), the pelleted tissue was reconstituted in ice-cold HBSS with 10% FCS and dispensed into culture dishes. Intact islets were manually collected under fluorescence visualisation using a Leica M205 FCA stereomicroscope (Leica Microsystems, Germany).

### RNA extraction and quantitative RT-qPCR

Total RNA from Ins-1 cells, pancreatic islets, and larval tissues was extracted using the RNA Simple Total RNA Extraction Kit (Tiangen, China). Complementary DNA (cDNA) was synthesised using the FastKing RT System (Tiangen, China), including genomic DNA removal. Transcript quantification was implemented via SYBR Green-based real-time PCR (Solarbio, China), with relative expression levels determined through the 2^−ΔΔCt^ method and normalised to Ct values of 18S rRNA in the control sample. A pre-experiment simultaneously analysed the expression levels of 18S and β-actin candidate reference genes, and 18S was selected for its high abundance and stable Ct values. Oligonucleotide sequences for amplification are provided in Supplementary Table 1 (see section on Supplementary materials given at the end of the article).

### Western blot

Cells were washed with phosphate-buffered saline (PBS) at 4°C and lysed in RIPA lysis buffer (Sigma-Aldrich, R0728, USA) supplemented with a proteolytic enzyme suppression cocktail (MCE, HY-K0010, USA) and phosphatase blockade agents (MCE, HY-K0021, USA). Following high-speed centrifugation (13,400 *g*, 10 min, 4°C), clarified lysates were quantified using a BCA assay (Thermo Fisher Scientific, A23228, USA). Protein aliquots underwent electrophoretic separation by SDS-polyacrylamide gels and electroblotting, transferred to PVDF membranes (Roche, Switzerland). Membranes were probed with antibodies specified in Supplementary Table 2. Chemiluminescent signals were visualised using a ChemiDoc™ XRS + System (Bio-Rad, 733BR2378, USA), and band intensities were quantified using ImageJ software (NIH, USA).

### Immunofluorescence

Ins-1 cells were cultured on coverslip substrates before genetic modification. Cells were fixed with 4% paraformaldehyde (PFA) (Merck, USA) for 15 min at room temperature for immobilisation. Permeabilisation and blocking were performed using PBS containing 5% FBS (Gibco, USA) and 0.1% Tween-20 (Solarbio, China) for 2 h at ambient temperature. Specimens were then probed with target-specific primary antibodies (diluted in permeabilisation/blocking buffer) and incubated overnight (16 h) at 4°C, followed by exposure to fluorophore-conjugated secondary antibodies for 2 h under ambient conditions (Supplementary Table 3). Nuclei were counterstained using DAPI-Fluoromount-G™ mounting medium (Yeasen Biotech, 36308ES11, China), and images were captured by fluorescence microscopy (Leica, SP8, Germany).

### O_2_ consumption analysis

Ins-1 cells were plated in 10 cm culture dishes and allowed to adhere before experimental treatment. Cells were enzymatically dissociated, pelleted by centrifugation (300 *g*, 5 min), and reconstituted in 1 mL of complete growth medium. The cell suspension was transferred to a 5 mL conical tube, adjusted with 2 mL of fresh medium, and vortexed for homogeneity. Cellular density was quantified by loading 10 μL of the mixture onto a haemocytometer for manual enumeration under phase-contrast microscopy, ensuring a standardised concentration of 1 × 10^6^ cells/mL.

Mitochondrial respiratory profiling was performed according to the manufacturer’s protocols for extracellular flux analysis. Sequential administration of metabolic modulators – oligomycin (ATP synthase inhibitor), FCCP (uncoupling agent), and antimycin A (complex III inhibitor) (APE×BIO, USA) – was executed through automated injector systems. Real-time oxygen consumption rates (OCR) were recorded and analysed using instrument-specific software post-calibration.

### ADP/ATP measurement

For cells cultured in 96-well plates, the ADP/ATP Ratio Assay Kit (BioAssay Systems, USA) was used. After aspirating the culture medium, 90 μL of ATP reagent was added to each well and mixed thoroughly. Following a 1 min incubation, luminescence (A) was measured using a multifunctional microplate reader. The plate was then incubated for 10 min while a fresh ADP reagent was prepared. After this period, luminescence was measured again (B). Subsequently, 5 μL of ADP reagent was immediately added to each well and mixed gently by tapping. After an additional 1 min incubation, luminescence (C) was measured. The ADP/ATP ratio was calculated using the formula: ADP/ATP = (C−B)/A.

### Transmission electron microscopy

Following experimental treatments, Ins-1 cells were fixed in 2.5% glutaraldehyde for 30 min at 25°C and stored at 4°C for 16 h. Cellular aggregates were consolidated by combining 200 μL of 20% bovine serum albumin with the suspensions and pelleting at 2,000 *g* for 5 min at room temperature, followed by resuspension in PBS.

Wild-type and *hnf1a^+/−^* larval specimens underwent parallel processing, including primary fixation in 2.5% glutaraldehyde (16 h, 4°C). Pancreatic islets were microdissected under fluorescence guidance (Leica M205 FCA system, Germany) and embedded in 1% low-melting-point agarose matrices. Ultrastructural analysis was conducted using a Hitachi HT-7800 TEM (Hitachi, Japan) operated at an 80 kV accelerating voltage.

### Statistical analysis

Quantitative analyses were executed using GraphPad Prism 8 (GraphPad Software Inc., USA) with parametric hypothesis-testing frameworks. Intergroup differences were assessed using two-sided unpaired *t*-tests, while multi-group comparisons were evaluated with one-way ANOVA. A significance threshold of *P* < 0.05 was applied. Data are presented as arithmetic mean ± SEM. Experimental replication metrics (biological/technical replicates) are explicitly annotated in the respective figure captions.

## Results

### *HNF1α*-Q125ter variant induced β-cell mitochondrial structure damage

**Figure 1:**
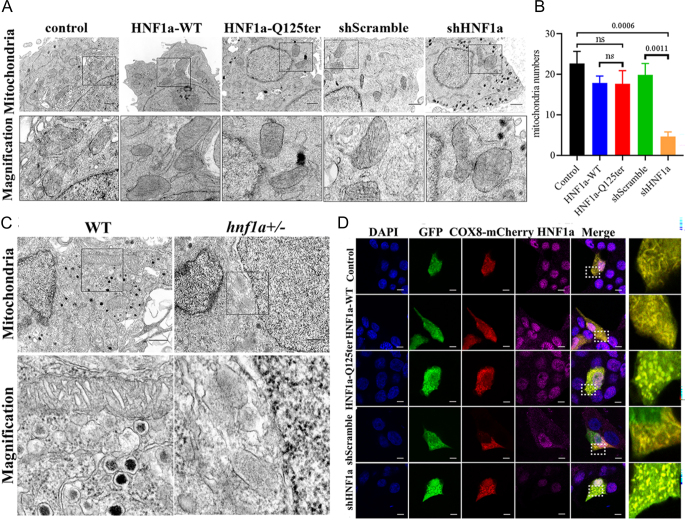
*HNF1α*-Q125ter induces mitochondrial ultrastructural abnormalities (A) Comparative TEM analysis of mitochondrial morphology across experimental groups (control, HNF1α-WT, HNF1α-Q125ter, shScramble, shHNF1α) 24 h post-transfection. Enlarged views demonstrate cristae disorganisation. Scale bars: 2 μm. Biological replicates: *n* = 5. (B) Quantitative assessment of mitochondrial integrity by counting the number. (C) TEM ultrastructural profiling of pancreatic β-cell mitochondria in WT and *hnf1a^+/−^* zebrafish at 6 dpf. Inset panels highlight cristae fragmentation (black box). Scale bars: 1 μm. (D) Confocal microscopy of COX8-mCherry-labelled mitochondrial networks in plasmid-transfected Ins-1 cells. Fluorescent markers: HNF1α (magenta), plasmid-transfected cells (GFP), mitochondrial COX8 (red), nuclei (DAPI). Scale bars: 25 μm. Statistical significance: one-way ANOVA; ns, no significance. Data presented as mean ± SEM. Biological replicates: *n* = 3. All independent experimental replicates ≥3, unless otherwise indicated. WT, wild type; TEM, transmission electron microscope. A full colour version of this figure is available at https://doi.org/10.1530/JME-25-0033.

To investigate the functional impact of the *HNF1α*-Q125ter variant, we generated a zebrafish line with HNF1α carrying a comparable mutation using CRISPR/Cas9 (Chen *et al.* 2022, Huang & Chen 2025). A previous study demonstrated that the *HNF1α*-Q125ter variant impairs pancreatic β-cell function and induces β-cell ER stress (Chen *et al.* 2022). During further analysis of the mechanisms underlying this ER stress, TEM revealed that *HNF1α*-Q125ter expression was associated with marked mitochondrial structural damage. To explore this effect in detail, we overexpressed *HNF1α*-Q125ter or induced a short hairpin RNA targeting HNF1α (*shHNF1α*) plasmid in the β-cell line Ins-1 832/13 β-cells. TEM revealed substantial disruption of mitochondrial cristae in *HNF1α*-Q125ter-expressing cells (Fig. 1A). However, mitochondrial number did not differ significantly compared with *HNF1α*-WT (Fig. 1B). Consistent with these findings, *hnf1a^+/−^* zebrafish also exhibited pronounced cristae damage (Fig. 1C). Immunostaining further confirmed fragmented mitochondrial cristae structure in both *HNF1α*-Q125ter and shHNF1α cells (Fig. 1D). Collectively, these results indicate that HNF1α-Q125ter induces mitochondrial structural damage *in vivo* and *in vitro*.

### *HNF1α*-Q125ter variant led to β-cell mitochondrial dysfunction

**Figure 2:**
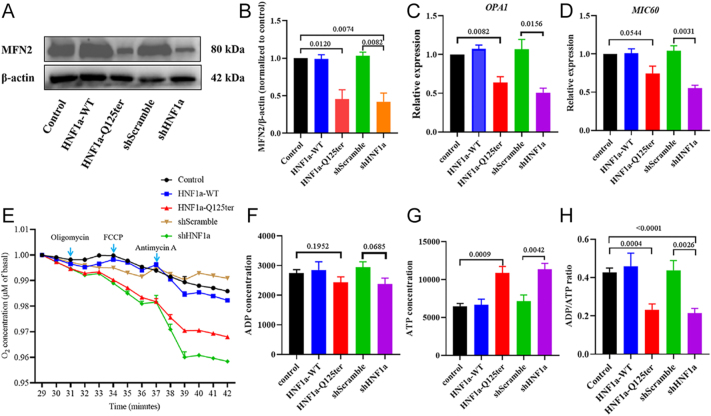
*HNF1α*-Q125ter triggers mitochondrial metabolic impairment. (A and B) Immunoblot profiles (A) and quantification analysis (B) of MFN2 (mitochondrial membrane remodelling machinery) in plasmid-transfected Ins-1 cells. β-actin-normalised protein abundance is shown. (C and D) Transcript quantification of *OPA1* (C) and *MIC60* (D) mRNA abundance in transfected Ins-1 cells. (E) Mitochondrial respiratory parameters measured via extracellular flux analysis. Oxygen consumption rates (OCR, pmol/min/μg protein) were recorded under basal conditions and after sequential pharmacological challenge: ATP synthase inhibitor (oligomycin, 2 μM), uncoupling agent (FCCP, 0.5 μM), and complex III inhibitor (antimycin A, 1 μM). Non-mitochondrial OCR was subtracted using the antimycin A baseline. Representative kinetic trace shown. (F) ADP concentration. (G) ATP concentration. (H) Bioenergetic profiling of intracellular nucleotide ratios. ADP/ATP ratios were calculated following quantification via bioluminescent assay (control vs HNF1α-WT/HNF1α-Q125ter/shScramble/shHNF1α). Data expressed as mean ± SEM; statistical methods: one-way ANOVA. Biological replicates: *n* ≥ 3 independent experiments, unless otherwise indicated. WT, wild type. A full colour version of this figure is available at https://doi.org/10.1530/JME-25-0033.

To better understand the impact of *HNF1α*-Q125ter on mitochondria, we assessed multiple aspects of mitochondrial function. We evaluated the effect of *HNF1α*-Q125ter overexpression on mitochondrial dynamics, mitochondrial content, mitochondrial oxygen consumption, and mitochondrial energy metabolism in Ins-1 cells. As illustrated in Fig. 2A and B, the protein level of mitofusin 2 (MFN2), a key mitochondrial fusion protein, was decreased in *HNF1α*-Q125ter-overexpressing cells, while it remained stable in *HNF1α*-WT-overexpressed cells. Consistently, the mRNA expression of several key mitochondrial cristae structure genes (*OPA1* (optic atrophy 1) and *MIC60* (MICOS complex subunit Mic60)) in the regulation of mitochondrial function was decreased (Fig. 2C and D). Over time, oxygen consumption per minute was markedly lower in *HNF1α*-Q125ter- and *shHNF1a*-expressing cells compared with controls or *HNF1α*-WT cells (Fig. 2E). To further evaluate mitochondrial capacity, we measured the ADP/ATP ratio to clarify the regulation of *HNF1α*-Q125ter on mitochondrial capacity metabolism. Compared with *HNF1α*-WT, the level of ADP was reduced in *HNF1α*-Q125ter (Fig. 2F). In contrast, the level of ATP was increased in *HNF1α*-Q125ter (Fig. 2G). Consequently, the ADP/ATP ratio was decreased in *HNF1α*-Q125ter (Fig. 2H). Together, these findings indicate that Ins-1 cells transfected with *HNF1α*-Q125ter exhibit impaired mitochondrial energy metabolism, suggesting mitochondrial functional impairment.

### *HNF1α*-Q125ter variant impaired mitophagy

**Figure 3:**
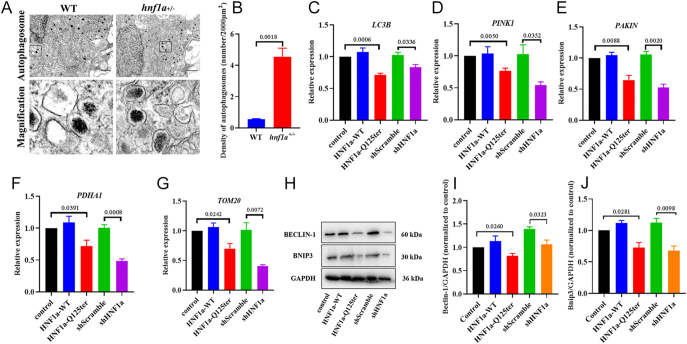
*HNF1α*-Q125ter variant impaired autophagy and mitophagy of β-cell. (A) Ultrastructural evidence of autophagic vacuole formation in WT and *hnf1a^+/−^* zebrafish β-cells at 6 dpf. Scale bars: 2 μm. Inset panels highlight autophagic vacuole formation (black box). (B) Quantitative density of autophagosomes. Analysis criteria: ≥6 spatially distinct fields per cell; biological replicates: WT (*n* = 3), *hnf1a^+/−^* (*n* = 3). Statistical significance determined by unpaired two-tailed *t*-test. (C) The mRNA levels of LC3B, a marker for autophagy. (D, E, F, G) The mRNA expression of several key mitophagy proteins, including PINK1, PAKIN, PDHA1, and TOM20. (H) Immunoblot profiles of autophagy regulators BECLIN-1 (autophagosome nucleation) and BNIP3 (mitochondrial cargo recognition) in transfected Ins-1 cells. (I and J) Densitometric quantification of BECLIN-1 (I) and BNIP3 (J) normalised to housekeeping protein GAPDH. Statistical significance determined by one-way ANOVA. Data expressed as mean ± SEM; statistical methods: zebrafish analysis: two-sided unpaired *t*-test; Ins-1 cell studies: one-way ANOVA; minimum independent replicates: 3 (technical replicates ≥6 for ultrastructural counts). WT, wild type. A full colour version of this figure is available at https://doi.org/10.1530/JME-25-0033.

Previous studies demonstrated that the *HNF1α*-Q125ter variant induces ER stress and activates nuclear factor erythroid 2-related factor 2 (Nrf2) transcription levels in zebrafish and Ins-1 cells (Chen *et al.* 2022). Nrf2 plays an important role in maintaining mitochondrial integrity under stress conditions (Dinkova-Kostova & Abramov 2015) and is also implicated in mitochondrial autophagy (Athale *et al.* 2012). Mitophagy, a specialised form of autophagy, removes damaged or unnecessary mitochondria. Based on our findings, we hypothesised that defective mitophagy might contribute to the observed mitochondrial structural abnormalities. TEM revealed fewer autophagosomes in *hnf1a^+/−^* zebrafish compared with controls (Fig. 3A). We counted the density of autophagosomes and found significantly decreased autophagic vacuole formation in *hnf1a^+/−^* (Fig. 3B). In addition, the mRNA levels of the autophagy marker, microtubule-associated protein 1 light chain 3β (LC3B), were decreased (Fig. 3C). *HNF1α*-Q125ter also suppressed the mRNA expression of key mitophagy-related genes, including PTEN-induced kinase 1 (PINK1), pyruvate dehydrogenase E1 subunit α1 (PDHA1), translocase of outer mitochondrial membrane 20 (TOM20), and Parkin RBR E3 ubiquitin-protein ligase (Parkin) (Fig. 3D, E, F, G). In addition, *HNF1α*-Q125ter inhibited mitophagy by suppressing the protein levels of BECLIN-1 and BNIP3 in Ins-1 cells (Fig. 3H, I, J). Collectively, these results indicate that *HNF1α*-Q125ter impairs autophagy and mitophagy.

### The possible mechanisms for *HNF1α*-Q125ter variant impaired mitophagy

**Figure 4:**
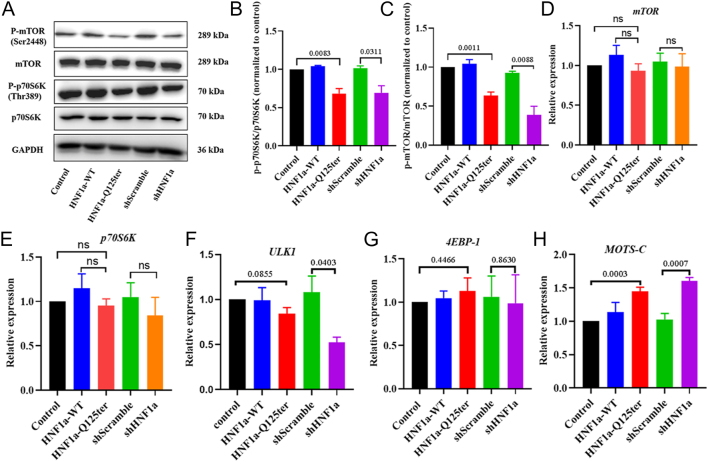
*HNF1α*-Q125ter-impaired autophagy and mitophagy might be through suppressing the mTOR/p70S6K signalling pathway. (A) Phosphorylation status profiling of mTOR pathway components in transfected Ins-1 cells. Immunoblots depict inhibited p-mTOR and its downstream effector p-p70S6K. Representative Western blot images of p-mTOR, mTOR, p70S6K, and p-p70S6K in different plasmid-transfected Ins-1 cells. (B and C) Kinase activation ratios quantified as p-p70S6K/p70S6K (B) and p-mTOR/mTOR (C). Data normalised to vector control baseline. (D, E, F, G, H) Transcript abundance of mTOR (D), p70S6K (E), ULK1 (F), 4EBP-1 (G), and MOTS-c (H) genes across experimental groups. mRNA levels determined by RT-qPCR with 18S rRNA normalisation. Statistical methods: one-way ANOVA; data representation: mean ± SEM from ≥3 biological replicates, unless otherwise indicated. WT, wild type. A full colour version of this figure is available at https://doi.org/10.1530/JME-25-0033.

To investigate the mechanism by which *HNF1α*-Q125ter affects autophagy and mitophagy, we examined the autophagy signalling pathway. Remarkably, we found that the mTOR/p70S6K autophagy signalling pathway was inhibited, as the protein levels of p-mTOR(Ser2448) and p-p70S6K(Thr389) were decreased in *HNF1α*-Q125ter-expressing Ins-1 cells (Fig. 4A, B, C). Concurrently, transcription levels of mTOR and p70S6K remained largely unchanged in *HNF1α*-Q125ter-expressed Ins-1 cells (Fig. 4D and E). Expression of downstream genes 4EBP-1 and ULK in p-mTOR(Ser2448) was also unaffected (Fig. 4F and G). However, the transcription levels of MOTS-c were increased in *HNF1*α-Q125ter-overexpressing Ins-1 cells (Fig. 4H). Concurrently, one study confirmed that overexpression of MOTS-c downregulated the level of phosphorylation of the mTORC1-related signalling pathway in β-cells (p-mTOR2448 and p-P70S6K1) (Kong *et al.* 2025). Taken together, these findings suggest that *HNF1a*-Q125ter may induce the upregulation of MOTS-c expression, thereby inhibiting the phosphorylation of the mTORC1-related signalling pathway in β-cells, and ultimately leading to impaired autophagy and mitophagy.

## Discussion

Heterozygous carriers of the HNF1α mutation experience a gradual decline in β-cell function, typically leading to diabetes onset in early adulthood (Ilharco & Silva Nunes 2018). Previous studies have emphasised key links between mitochondrial activity and β-cell functionality (El-Assaad *et al.* 2010, Kwak & Park 2016). Notably, structural and functional impairments of β-cell mitochondria have been associated with insulin secretory defects in both diabetic patients and insulin-resistant iPSCs (Anello *et al.* 2005, Burkart *et al.* 2016). However, the role of the new variant *HNF1α*-Q125ter in regulating mitochondrial metabolism within β-cells remains unclear.

Mitochondrial metabolism plays a pivotal role in β-cells, particularly in coupling extracellular glucose through the generation of ATP. Disruptions of mitochondrial oxidative metabolism can have significant consequences (Mulder 2017, Nicholas *et al.* 2017). Furthermore, mitochondria are crucial for maintaining β-cell mass. Consequently, mitochondrial dysfunction can alter membrane potential, potentially triggering apoptosis (Xiong *et al.* 2014). Collectively, these findings underscore the importance of mitochondria in β-cell impairment and diabetes pathogenesis.

In this study, we demonstrated that *HNF1α*-Q125ter induces mitochondrial dysfunction in both the *hnf1a^+/−^* zebrafish model and *HNF1α*-Q125ter-overexpressing Ins-1 cells. Mitochondrial dynamics, including fusion, fission, and mitophagy, are essential for maintaining mitochondrial quality and function (Yu *et al.* 2020, Poderoso *et al.* 2022, Gambardella *et al.* 2023). We found that *HNF1α*-Q125ter compromised mitochondrial structure, morphology, and content were compromised in *hnf1a^+/−^* and *HNF1α*-Q125ter-overexpressing Ins-1 cells. Furthermore, *HNF1α*-Q125ter decreased the mitochondrial ADP/ATP ratio. This finding is contrary to a previous study in mouse β-cells, where the most common *HNF1α* mutation in MODY3 (P291fsinsC) reduced mitochondrial ATP production (Wang *et al.* 2000). Collectively, these observations suggest that *HNF1α*-Q125ter plays a significant role in ATP production, primarily within the mitochondria.

We also found that *HNF1α*-Q125ter impairs mitochondrial respiration. This finding aligns with Cardenas-Diaz *et al.*, who reported that loss of *HNF1α* in human embryonic stem cell-derived β-cells also impaired mitochondrial respiration and decreased levels of *LINKA*, a human-specific long non-coding RNA related to *HNF1α* (Cardenas-Diaz *et al.* 2019). Taken together, these results strongly suggest the involvement of *HNF1α*-Q125ter in regulating mitochondrial function.

Another study indicated that *Pdx1*, a gene associated with MODY, regulates mitochondrial function by transcriptionally controlling mitophagy in pancreatic β-cells (Soleimanpour *et al.* 2015). However, the relationship between *HNF1α*-MODY3 and mitophagy dysfunction in β-cells has remained unclear. In our study, we observed reduced transcriptional and protein levels of mitophagy-related genes in the presence of *HNF1α*-Q125ter. *In vivo*, we also observed a reduction in the number of autophagic vacuole formation in pancreatic β-cells of the *hnf1a^+/−^* zebrafish model. These results suggest that *HNF1α*-Q125ter plays a crucial role in mitophagy within β-cells. Subsequently, we delved deeper into the mechanism underlying *HNF1α*-Q125ter regulation.

The mTOR pathway is known to influence mitophagy (Liu *et al.* 2021, Zheng *et al.* 2021, Zhao *et al.* 2022). For instance, Zhao *et al.* (2022) reported that leonurine activates mitophagy to protect bone mesenchymal stem cells from oxidative stress-induced damage by inhibiting the PI3K/Akt/mTOR pathway. Similarly, Zheng *et al.* (2021) demonstrated that rapamycin inhibits apoptosis and enhances mitophagy in neuronal cells via PI3K/AKT/mTOR blockade. Despite this evidence, the specific role of mTOR signalling in *HNF1α*-Q125ter-mediated mitophagy had not been defined.

Our findings revealed reduced levels of p-mTOR(Ser2448) and p-p70S6K(Thr389) in *HNF1α*-Q125ter-overexpressing cells. These findings suggest that *HNF1α*-Q125ter impairs autophagy and mitophagy in β-cells, at least in part, through inhibition of the mTOR/p70S6k pathway.

However, this study has some limitations. First, the *in vivo* and *in vitro* models were limited to zebrafish and non-human β-cell lines. Second, because autophagy and mitophagy are dynamic processes, their impairment is best evaluated with multiple complementary approaches, including immunoblotting, TEM, and immunofluorescence-based colocalisation or mitophagy flux analysis. Future studies should examine *HNF1α*-Q125ter-mediated autophagy and mitophagy in pancreatic islets from diabetic patients to validate these findings.

## Conclusions

**Figure 5:**
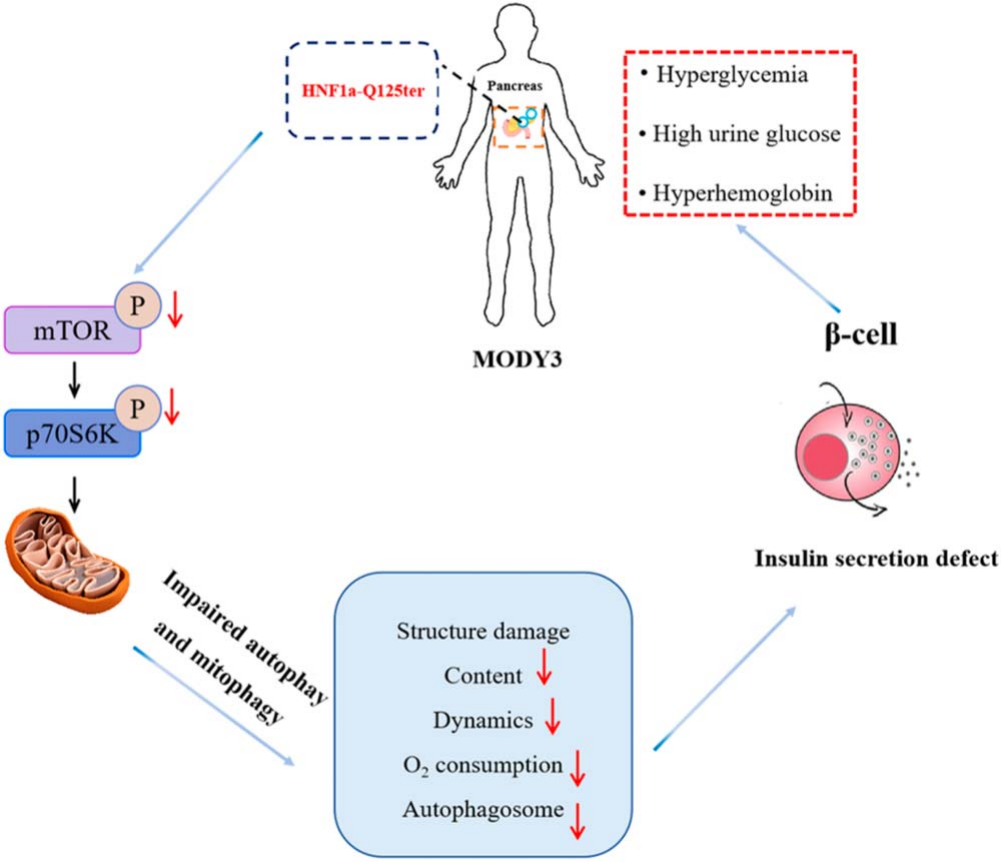
Mechanistic framework of *HNF1α*-Q125ter-driven β-cell mitochondrial dysfunction in MODY3 pathogenesis. This schematic delineates the pathogenic cascade by which the HNF1α-Q125ter truncation variant disrupts pancreatic β-cell mitochondrial homoeostasis. The mutation impairs autophagic clearance of dysfunctional mitochondria (mitophagy) via suppression of the p-mTOR(Ser2448)/p-p70S6K(Thr389) signalling axis (phosphorylation-dependent inactivation). These defects culminate in multilevel mitochondrial compromise: structural degeneration – cristae disorganization and membrane integrity loss; biogenesis suppression – reduced mitochondrial mass and replication capacity; and dynamic imbalance – fragmented network architecture with impaired fusion – fission equilibrium, and quality control failure – accumulation of depolarised organelles due to decreased autophagic vacuole formation. This integrated mechanism drives the progressive β-cell mitochondrial dysfunction characteristic of MODY3 progression. A full colour version of this figure is available at https://doi.org/10.1530/JME-25-0033.

Our study suggests that *HNF1α*-Q125ter impairs mitophagy in β-cells by inhibiting the p-mTOR(Ser2448)/p-p70S6k(Thr389) pathway, potentially contributing to sulfonylurea insensitivity in patients with *HNF1α*-Q125ter-MODY3 (Fig. 5). These findings provide a foundation for future research into therapeutic strategies aimed at protecting β-cell mitochondria by modulating the mTOR pathway, potentially offering new treatment avenues for sulfonylurea-insensitive MODY3 patients.

## Supplementary materials



## Declaration of interest

The authors have no conflicts of interest to declare.

## Funding

This work was supported by grants from the Natural Science Foundation of Zhejiang Provincehttps://doi.org/10.13039/501100004731, China (LQ24H070007), and the Medical Science and Technology Project of Zhejiang Provincehttps://doi.org/10.13039/501100017594 (2025KY1577).

## Author contribution statement

YC and XZ substantially contributed to the conception and the design of the study. JH and XC were responsible for the acquisition, analysis, and interpretation of the data. FJ contributed to manuscript drafting and critical revisions of the intellectual content, and approved the final manuscript to be published. All authors read and approved the final manuscript.
