# Supplementary material for: HNF1α-Q125ter-mediated mitochondrial dysfunction and impaired mitophagy in β-cells
Source: J Mol Endocrinol. 2025 Nov 10;75(4):e250033. doi: 10.1530/JME-25-0033 (PMC12910567; doi:10.1530/JME-25-0033)
Supplement: Supplementary file 1 [file supplementary_materials.pdf]

Supplementary Table 1. Primer sequence for RT-qPCR used in this study

**Gene**	**Forward primers (5' to 3')**	**Reverse primers (5' to 3')**
18s	TCCGATAACGAACGAGAC	CTAAGGGCATCACAGACC
PINK1	CCCAAGCAACTAGCCCCTC	GGCAGCACATCAGGGTAGTC
PAKIN	CCCACCTCTGACAAGGAAACA	TCGTGAACAAACTGCCGATCA
mTOR	ACTGGAGGCTGATGG ACACA	GGCTCTCCAAGT TCCACACC
p70S6K	CATCGGCACCACTTCCAATA	TTCATACGCAGGTGCTCTGG
TOM20	GGTACTGCATCTACTTCGACCG	TGGTCTACGCCCTTCTCATATTC
PDHA1	ATGGAATGGGAACGTCTGTTG	CCTCTCGGACGCACAGGATA
LC3B	GATGTCCGACTTATTCGAGAGC	TTGAGCTGTAAGCGCCTTCTA
OPA1	CAGCTGGCAGAAGATCTCAAG	CATGAGCAGGATTTTGACACC
MIC60	AGGGAGACACTCCAGCTTCA	GACGAGCTGCAACTTCTTCG
ULK1	GGCAAGTTCGAGTTCTCCCG	TAATGCACTTGACGGCGACC
4EBP-1	GAAGTTGCTCTACCCAGTGTCC	GATAGCCGTTCCTTTCATTTGG
MOTS-c	ATACCGCCATCTTCA	ATCCTCCTTAGTCCTTT

Supplementary Table 2. Primary antibody species in Western blotting used in this study.

**Antibodies**	**Brand**	**No.**
β-actin	Cell Signaling Technology	3700
GAPDH	Cell Signaling Technology	2118
MFN2	Abcam	ab124773
BNIP3	Abcam	ab10433
BECLIN-1	Cell Signaling Technology	3738
mTOR	Cell Signaling Technology	2972
p-mTOR (Ser2448)	Cell Signaling Technology	2971
p70S6K	Cell Signaling Technology	2708
p-p70S6K (Thr389)	Cell Signaling Technology	9234
Goat anti-Rabbit IgG (H+L) Secondary Antibody	ThermoFisher Scientific	31460
Goat anti-Mouse IgM Secondary Antibody	ThermoFisher Scientific	31440

Supplementary Table 3. Antibodies of immunofluorescence staining

**Antibodies**	**Brand**	**No.**
HNF1a	Cell Signaling Technology	89670
Alexa Fluor^{TM} 647 goat anti-rabbit IgG (H+L)	ThermoFisher Scientific	A21244
